# Cytocompatibility of PMMA and Titanium Boston Keratoprosthesis Backplates with Human Corneal Fibroblasts

**DOI:** 10.3390/bioengineering13050517

**Published:** 2026-04-29

**Authors:** Antonio Esquivel Herrera, Liangju Kuang, Mark Krauthammer, Michael Bednar, Eleftherios I. Paschalis, Thomas H. Dohlman

**Affiliations:** 1 Schepens Eye Research Institute of Mass Eye and Ear, 20 Staniford Street, Boston, MA 02114, USA; aesquivelherrera@meei.harvard.edu (A.E.H.); liangju_kuang@meei.harvard.edu (L.K.); mkrauthammer@meei.harvard.edu (M.K.); mbednar@meei.harvard.edu (M.B.); eleftherios_paschalis@meei.harvard.edu (E.I.P.); 2 Department of Ophthalmology, Harvard Medical School, 243 Charles Street, Boston, MA 02114, USA

**Keywords:** Boston Keratoprosthesis, polymethyl methacrylate (PMMA), titanium, corneal fibroblasts, retroprosthetic membrane, myofibroblast differentiation, mechanotransduction, focal adhesion kinase, α-smooth muscle actin, biomaterial cytocompatibility

## Abstract

This study evaluates how titanium and polymethyl methacrylate (PMMA) Boston Keratoprosthesis backplate substrates influence human corneal fibroblast proliferation, cytotoxicity, morphology, activation phenotype, and mechanotransductive signaling. Human corneal fibroblasts were cultured on titanium and PMMA, with tissue culture plastic or glass as controls. Proliferation was assessed over 7 days using metabolic assays, and cytotoxicity was measured by lactate dehydrogenase release. Cell morphology and surface coverage were examined by scanning electron microscopy, while immunofluorescence quantified fibroblast-specific protein 1 (FSP-1) and α-smooth muscle actin (α-SMA). Gene expression of α-SMA, collagen I, FSP-1, and focal adhesion kinase (FAK) was analyzed by quantitative PCR. Cells cultured on both substrates maintained stable viability with modest increases in estimated cell numbers and comparable proliferation curves, indicating preserved metabolic activity without growth suppression. Cytotoxicity remained low and similar between groups. SEM demonstrated broader and more continuous cell spreading on titanium, whereas cells on PMMA were more sparsely distributed. Immunofluorescence showed higher FSP-1 expression on titanium and increased α-SMA on PMMA. Gene expression analysis revealed higher FAK transcripts on PMMA, with no significant differences in α-SMA, FSP-1, or collagen I. These results confirm the cytocompatibility of both titanium and PMMA backplates with human corneal fibroblasts and support their use with the Boston Keratoprosthesis.

## 1. Introduction

The Boston Keratoprosthesis (BKPro) has transformed the management of severe corneal blindness in patients who are poor candidates for conventional penetrating keratoplasty (PK) [1]. The BKPro features a collar-button design in which carrier corneal tissue is sandwiched between the BKPro frontplate and backplate, with the KPro optical stem passing through the carrier corneal tissue [2]. The BKPro type I backplate is currently manufactured from either polymethyl methacrylate (PMMA) or titanium. Both materials are clinically accepted and considered cytocompatible [3,4,5].

Biocompatibility between the BKPro and host corneal tissue is essential for long-term device success. Effective integration requires a balanced wound-healing response at the device–tissue interface that preserves stromal integrity while limiting chronic inflammation and aberrant extracellular matrix remodeling. Disruption of this equilibrium can compromise device retention and predispose to complications, including corneal melt, infection, and retroprosthetic membrane (RPM) formation [2,6,7,8,9].

RPM is the most common postoperative complication after BKPro implantation [7,8,10,11]. Histopathologic studies describe RPM as a fibrocellular membrane composed of activated stromal fibroblasts and extracellular matrix, implicating myofibroblast differentiation and dysregulated wound healing at the device–tissue interface [12]. These observations highlight the importance of device–tissue interactions in shaping stromal cell behavior and influencing structural stability and long-term outcomes after BKPro surgery.

Corneal fibroblasts are highly responsive to their microenvironment [13]. When stressed, injured or exposed to profibrotic cytokines, they shift toward activated states characterized by changes in adhesion, cytoskeletal structure, and expression of mesenchymal and contractile markers, such as FSP-1 and α-smooth muscle actin (α-SMA) [2,13]. Since fibroblasts interpret mechanical signals from their substrate alongside biochemical cues, the material composition of the backplate may influence their activation profile [2,13,14,15].

While previous studies have evaluated BKPro cytocompatibility by comparing human corneal epithelial cells on PMMA vs. titanium backplates and human corneal fibroblasts (hCFs) on various titanium backplate topographies [4,5], the extent to which backplate material properties independently influence corneal fibroblast behavior remains incompletely understood.

We hypothesized that titanium and PMMA substrates differentially modulate corneal fibroblast phenotype, including activation state and expression of mechanotransduction-associated markers, under controlled in vitro conditions.

To test this, we evaluated hCF metabolic activity, cytotoxicity, morphology, and expression of activation and mechanosensitive markers, as well as gene expression, under baseline conditions. These analyses were designed to assess material-dependent cellular responses relevant to biocompatibility and stromal remodeling, with potential implications for BKPro-associated complications such as RPM formation.

## 2. Materials and Methods

### 2.1. Material Preparation

Medical-grade PMMA and titanium discs of 13 mm diameter and 0.510 mm thickness were used to model the Boston Keratoprosthesis backplate material and surface topography in a cell culture system. Titanium backplates were surface-finished using 150 WP Aluminum Oxide sandblasting media in an identical process and with similar parameters as the titanium BKPro backplate that is clinically available. Likewise, PMMA discs were processed in the same manner as PMMA BKPro devices. Discs were cleaned and sterilized in the same manner as clinically available BKPro devices.

### 2.2. Cell Culture and Seeding

Immortalized human corneal fibroblasts (hCFs; Applied Biological Materials, Richmond, BC, Canada; Cat. #T0578) were maintained in DMEM supplemented with 10% fetal bovine albumin (FBS) and 1% antibiotic-antimycotic at 37 °C and 5% CO_2_. Cells were seeded at 2.0 × 10^4^ cells/well in 24-well plates containing one sterilized PMMA or titanium disc per well (final volume = 500 µL). Cells cultured on tissue culture polystyrene (TCPS) served as substrate controls for LDH, MTS, and qPCR assays, while glass slides served as substrate controls for scanning electron microscopy (SEM) and immunocytochemistry (ICC) readouts.

### 2.3. Cell Viability and Proliferation Kinetics (MTS Assay)

Metabolic activity, reflecting cell proliferation, was quantified at days 2, 5, and 7 using the CellTiter 96^®^ AQueous One Solution Cell Proliferation Assay (Promega, Madison, WI, USA; Cat. #G3580). An MTS/PMS mix was prepared per the manufacturer’s protocol at a 20:1 ratio. Medium was replaced with 500 µL fresh DMEM containing 10% FBS, and MTS/PMS was added; the mixture was incubated for 2 h at 37 °C, and 100 µL aliquots were read at 490 nm. The absorbance was converted into estimated cell numbers using a plate-matched logarithmic calibration curve generated from standards ranging from 20,000 to 150,000 cells per well on TCPS. Cell number estimation was based on a calibration curve generated under standardized culture conditions and applied uniformly across all substrates to enable relative comparisons between conditions. However, as substrate-dependent differences may influence MTS readouts in cellular metabolism or assay performance, these measurements are interpreted as relative estimates rather than absolute cell counts.

### 2.4. Cytotoxicity Analysis (LDH Assay)

Cytotoxicity was measured at days 2, 5, and 7 using the CytoTox 96^®^ Non-Radioactive Cytotoxicity Assay (Promega, Madison, WI, USA; Cat. #G1780) and following the manufacturer’s protocol. At each time point, 50 µL of culture supernatant was collected and transferred to a 96-well plate; 50 µL of LDH substrate mix was added, and the plate was incubated for 30 min at 37 °C in the dark. Stop solution was added, and absorbance was recorded at 490 nm using a microplate reader (Synergy™ MX, BioTek Instruments, Winooski, VT, USA; controlled using Gen5™ Microplate Reader Software, version 1.11.5). The kit-supplied positive control (LDH) was used to determine the cytotoxicity percentage using the following formula.

$$\%\mathrm{Cytotoxicity}=\frac{\left(\mathrm{Sample}-\mathrm{Blank}\right)}{(\mathrm{LDH}\;\mathrm{Positive}\;\mathrm{Ctrl}-\mathrm{Blank})}\times 100$$

### 2.5. Scanning Electron Microscopy (SEM)

To investigate hCF morphology on titanium, PMMA, and glass substrates, cells were seeded at a density of 2 × 10^4^ cells per substrate and incubated at 37 °C with 5% CO_2_. A control group on glass substrates was stimulated with TGF-β1 (100 ng/mL) for 48 h to induce myofibroblast differentiation. After seven days of incubation, the culture media was aspirated, and the samples were immediately immersed in freshly prepared 1/4 Karnovsky’s fixative in 0.1 M sodium cacodylate buffer (pH 7.4; ~2 mL per well) for 4 h in a chemical fume hood. The samples were then post-fixed with 1% osmium tetroxide in 0.1 M sodium cacodylate buffer. After fixation, the samples were dehydrated through a graded ethanol series (25%, 50%, 75%, 95%, and 100%) and then critical-point dried. Finally, the samples were mounted onto metal stubs, sputter-coated with gold, and imaged using a JCM-7000 NeoScope™ (JEOL Ltd., Tokyo, Japan) scanning electron microscope. To semi-quantitatively analyze cell surface coverage, 3 non-overlapping images per substrate were acquired from both the center and the periphery at 100× magnification using identical imaging parameters across all conditions. Surface coverage of cells on SEM micrographs was measured manually using ImageJ (v. 1.52). Cell boundaries were delineated using the freehand selection tool to define the regions of interest (ROIs). ROI selection was performed using a standardized and predefined approach applied uniformly across all samples to minimize selection bias. The cell surface coverage for each image (*n* = 3) was calculated as the area fraction, defined as the ratio of the manually traced cellular area to the total field-of-view area. The mean surface coverage from three images per substrate was then calculated and used for comparison, with values averaged to generate a single measurement per condition within each experiment.

### 2.6. Immunocytochemistry and Confocal Microscopy

hCFs were seeded at a density of 2.0 × 10^4^ per well and incubated at 37 °C with 5% CO_2_. A control group on glass substrates was stimulated with TGF-β1 to induce differentiation into human corneal myofibroblasts (hCMFs). After seven days of incubation, immunofluorescence staining was performed to assess levels of alpha-smooth muscle actin (α-SMA) and fibroblast-specific protein 1 (FSP-1). Briefly, cells were fixed with 4% freshly prepared paraformaldehyde, then washed three times with 1% Triton X-100 in PBS. Cells were then blocked with 1% BSA in PBS for 1 h at room temperature and incubated with a rabbit monoclonal anti-α-SMA antibody (ab124964, Abcam, Cambridge, UK; 1:200) or a rabbit monoclonal FSP1 antibody (S100A4, Abcam, Cambridge, UK; 1:200) for 1 h at room temperature, followed by overnight incubation in a humidified chamber. After washing with PBS three times, cells were incubated with Goat anti-Rabbit IgG (H + L) Cross-Adsorbed Secondary Antibody, Alexa Fluor™ 488 (A-11008, Invitrogen, Carlsbad, CA, USA; part of Thermo Fisher Scientific; 1:500) or Donkey anti-Rabbit IgG (H + L) Highly Cross-Adsorbed Secondary Antibody, Alexa Fluor™ 594 (A-21207, Invitrogen, Carlsbad, CA, USA; part of Thermo Fisher Scientific; 1:500) for 1 h at room temperature. The samples were then washed with PBS three times and mounted in VECTASHIELD containing 4′,6-diamidino-2-phenylindole (DAPI, Vector Laboratories, Newark, CA, USA). A confocal laser scanning microscope (TCS SP8, Leica Microsystems, Wetzlar, Germany; LAS X software, version LAS_X_Small_3.6.0_20104) was used for fluorescence imaging. To capture representative data, three distinct fields of view were selected from both the center and the periphery of each substrate during the imaging process. Mean fluorescence intensity (MFI) was quantified manually using ImageJ software (v. 1.52). Micrographs were first converted to 8-bit grayscale to standardize pixel depth across samples. To ensure objective segmentation within these regions, a fixed global thresholding protocol was applied (16 for α-SMA and 21 for FSP) to isolate the cellular signal from the background [16]. The Integrated Density (IntDen) was recorded for each ROI and analyzed.

### 2.7. RNA Isolation and Real-Time Quantitative PCR

On day 7, cells were collected using an RLT + BME 0.1% lysis buffer. Total RNA was extracted from hCFs using the RNeasy Mini Kit (QIAGEN, Hilden, Germany) according to the manufacturer’s instructions. RNA concentration was measured with a NanoDrop 2000 spectrophotometer (Thermo Fisher Scientific, Waltham, MA, USA). Complementary DNA was synthesized from the isolated RNA using the QIAGEN QuantiTect Reverse Transcription Kit. The expression of FSP-1, α-SMA, COL1A1, and FAK was quantified by real-time PCR using TaqMan Universal PCR Master Mix and specific TaqMan assays for human S100A4 (Assay ID Hs00243202_m1; Thermo Fisher Scientific), Acta2 (Assay ID Hs00426835_g1; Thermo Fisher Scientific), COL1A1 (Assay ID Hs00164004_m1; Thermo Fisher Scientific), PTK2 (Assay ID Hs01056457_m1; Thermo Fisher Scientific) and GAPDH (Assay ID Hs02786624_g1; Thermo Fisher Scientific) as the internal reference gene. A group of hCFs treated with recombinant TGF-β1 (100 ng/mL) (Peprotech, Rocky Hill, NJ, USA) served as a control to evaluate fibroblast to myofibroblast differentiation. Gene expression was normalized to GAPDH and reported as copies per 10^6^ GAPDH.

### 2.8. Statistical Analysis

All experiments were performed with three replicates per condition within each independent experiment. Independent experiments were repeated at least twice. Data from all experiments were pooled, yielding *n* = 6 observations per condition across two independent experiments. Results are expressed as mean ± standard deviation (SD), with the statistical unit of analysis defined as the averaged value per condition from each independent experiment, which were pooled for analysis. Statistical comparisons were conducted using Welch’s *t* test, unpaired t test, or two-way ANOVA followed by Tukey’s multiple-comparison test using GraphPad Prism 11. A *p*-value < 0.05 was considered statistically significant. The main comparisons were made between titanium and PMMA conditions, with control substrates (TCPS or glass) included as assay-specific references.

## 3. Results

### 3.1. Cell Proliferation Kinetics

**Figure 1:**
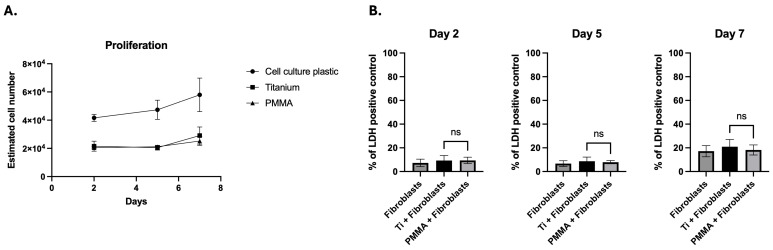
Cells grown on both Ti and PMMA showed stable metabolic activity over 7 days, with slight increases in estimated cell numbers and similar proliferation curves (Two-way ANOVA, *p* = 0.9) (**A**). This suggests maintained metabolic activity without growth suppression on either substrate. Additionally, LDH release (**B**) remained low and was comparable between the two materials at all time points (Welch’s *t* test, *p* = 0.6). Data are presented as mean ± standard deviation (SD). Each experiment included three technical replicates per condition, which were averaged, and data from independent experiments were combined for analysis (*n* = 6 per group, from two independent experiments). Statistical comparisons were performed between titanium (Ti) and polymethyl methacrylate (PMMA) conditions. ns, not significant (*p* ≥ 0.05).

hCFs were cultured on PMMA, titanium, or tissue culture polystyrene (TCPS) (control) substrates. Through 7 days of culture, estimated HCF cell numbers remained comparable in the PMMA and titanium groups. After 48 h of culture, the estimated cell number was similar in the titanium group as compared to the PMMA group (21,582 ± 3549 for titanium vs. 20,491 ± 861 for PMMA) (*p* = 0.9), as was the case at day 5 of culture (20,471 ± 1698 for titanium vs. 21,197 ± 516 for PMMA; *p* = 0.9). At day 7, the estimated cell number remained similar between groups (29,092 ± 6097 for titanium vs. 25,209 ± 3090 for PMMA; *p* = 0.4). When expressed as fold change, the estimated cell number in TCPS controls was approximately twofold higher than in both the PMMA and titanium groups at all time points. On day 2, TCPS-estimated cell numbers were 1.9× higher than those for titanium and 2.0× higher than those for PMMA (both *p* < 0.0001). This twofold difference was maintained at day 5 (2.3× vs. titanium; 2.2× vs. PMMA; *p* < 0.0001) and day 7 (1.9× vs. titanium; 2.3× vs. PMMA; *p* < 0.0001) (Figure 1A).

### 3.2. Cytotoxicity Assessment

The cytotoxicity of PMMA, titanium, or TCPS (control) against hCFs was evaluated on days 2, 5, and 7 using the LDH Cytotoxicity Assay. The positive control included with the kit validated the assay by generating the expected maximum LDH release. On day 2, both titanium and PMMA showed low cytotoxicity (9.3% ± 4.2% for titanium; 9.3% ± 2.7% for PMMA; *p* = 0.9), comparable to TCPS (7.3% ± 3.2%), indicating cytocompatibility at this early culture time point. By day 5, LDH release for both substrates remained within the non-cytotoxic range [17] (8.6% ± 3.5% for titanium; 7.8% ± 1.3% for PMMA), with no statistically significant difference between them (*p* = 0.6) and values comparable to the TCPS control (6.7% ± 2.4%). On day 7, LDH levels remained stable and low in both groups (20.9% ± 6.1% for titanium; 18.2% ± 4.2% for PMMA; *p* = 0.4), again approximating TCPS levels (17.2% ± 4.7%). These results confirmed that neither substrate induces overt metabolic stress or cell lysis (Figure 1B).

### 3.3. Scanning Electron Microscopy (SEM) Assessment of hCF Distribution

**Figure 2:**
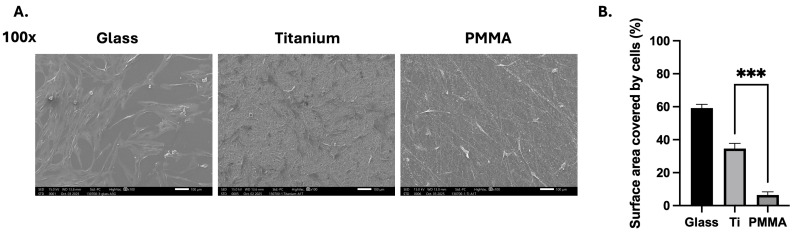
Scanning electron microscopy was used to evaluate cellular morphology and distribution across titanium and PMMA substrates. Representative images showed broader coverage on titanium, whereas PMMA surfaces exhibited a less uniform distribution with more exposed substrate (**A**), scale bar: 100 µm. Semi-quantitative analysis of surface area coverage (%) confirmed that a greater proportion of the titanium surface was occupied by cells than on PMMA (Welch’s *t* test, *p* = 0.0006). (**B**). Data are presented as mean ± standard deviation (SD) from three non-overlapping images per condition from independent experiments (*n* = 6 per group). Experiments were independently repeated at least twice with consistent trends, and the combined data from independent experiments are shown. Statistical comparisons were performed between Ti and PMMA conditions. *** *p* < 0.001.

Scanning electron microscopy (SEM) was used to evaluate hCF morphology, distribution and surface coverage on titanium and PMMA substrates. A glass substrate served as a control. Representative micrographs showed that hCFs adhered to both PMMA and titanium and exhibited typical elongated morphology with visible cytoplasmic extensions. Qualitative assessment revealed broader, more continuous cell spreading across titanium surfaces, whereas cells cultured on PMMA appeared more discretely distributed (Figure 2A). Semiquantitative analysis assessed these differences; the percentage of surface area covered by cells was measured for each substrate. HCFs cultured on titanium exhibited a significantly greater percentage of surface area coverage compared to those cultured on PMMA (34.6% ± 3.2% vs. 6.4% ± 1.9%; *p* = 0.0006). The control group showed an area coverage of 59.2% ± 2.3% (Figure 2B).

### 3.4. Immunocytochemistry and Confocal Microscopy of Fibroblast Activation and Myofibroblast Markers

**Figure 3:**
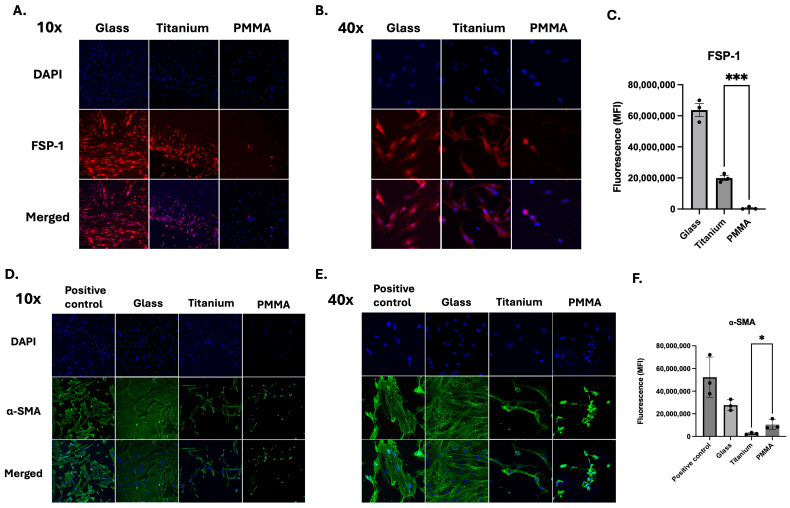
Immunohistochemical analysis at low (10×) and high (40×) magnification revealed substrate-dependent modulation of FSP-1 (red, **A**–**C**) and α-SMA (green, **D**–**F**) expression. Representative 10× images show overall staining distribution (**A**,**D**), while 40× images show cellular-level signal (**B**,**E**). Fluorescence intensity was quantified in 40× fields (*n* = 3 fields per condition) within a single experiment. Fibroblasts cultured on titanium exhibited significantly higher FSP-1 fluorescence intensity than those on PMMA (Unpaired *t* test, *p* = 0.0003) (**C**). Conversely, α-SMA expression was significantly higher in fibroblasts cultured on PMMA than on titanium (Unpaired *t* test, *p* = 0.0389). Cells cultured on a glass slide and stimulated with TGF-β1 served as a positive control (**F**). Data are presented as mean ± standard deviation (SD) from three non-overlapping images per condition (*n* = 3 per group). Statistical comparisons were performed between Ti and PMMA conditions. * *p* < 0.05, *** *p* < 0.001.

Immunofluorescence staining was performed to assess expression of FSP-1 (fibroblast-specific protein 1) and α-smooth muscle actin (α-SMA) in corneal fibroblasts cultured on titanium and PMMA substrates, with a glass substrate serving as a control. FSP-1 is a cytoplasmic protein associated with activated fibroblasts and cellular motility, whereas α-SMA is a contractile cytoskeletal protein that serves as a hallmark of myofibroblast differentiation and fibrotic remodeling [13]. Representative images were obtained at both 10× and 40× magnification to evaluate overall distribution and cellular-level expression patterns. At 10× magnification, hCFs cultured on titanium demonstrated broader cellular coverage with more uniform FSP-1 staining across the surface (Figure 3A). In contrast, hCFs cultured on PMMA showed relatively less cell coverage of the substrate with more prominent α-SMA signal within individual cells (Figure 3D). Quantification of fluorescence intensity was performed on 40× images under identical acquisition settings for all groups. FSP-1 staining demonstrated positive cytoplasmic expression in fibroblasts (Figure 3B). Quantitatively, cells on titanium appeared to exhibit more intense fluorescence compared to those on PMMA. Quantitative analysis confirmed a significantly higher FSP-1 fluorescence intensity (MFI) in fibroblasts cultured on titanium compared with PMMA (19,918,220 ± 2,742,651 for titanium; 620,160 ± 805,386 for PMMA; *p* = 0.0003) (Figure 3C). In contrast, α-SMA staining (Figure 3D) showed stronger expression in fibroblasts cultured on PMMA compared to titanium, with localization along the cytoskeleton in both groups (Figure 3E). Quantitative analysis demonstrated significantly higher α-SMA fluorescence intensity (MFI) in fibroblasts cultured on PMMA relative to titanium (10,515,582 ± 4,476,019 for PMMA; 2,573,460 ± 779,638 for titanium; *p* = 0.0389) (Figure 3F).

### 3.5. mRNA Expression of Fibroblast Activation and Myofibroblast Markers

**Figure 4:**
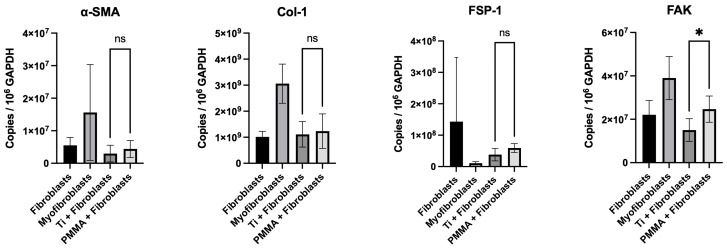
Quantitative PCR was used to assess absolute mRNA expression of α-SMA, collagen I, FSP-1, and FAK in fibroblasts cultured on titanium and PMMA substrates. Gene expression was normalized to GAPDH and reported as copies per 10^6^ GAPDH copies. Expression levels of α-SMA and collagen I were comparable between groups, with no statistically significant differences (Welch’s *t* test, *p* = 0.3 and *p* = 0.7, respectively). In contrast, FSP-1 showed a higher trend towards significance, and FAK mRNA expression was significantly higher in fibroblasts cultured on PMMA than on titanium (Welch’s *t* test, *p* = 0.06 and *p* = 0.01, respectively). Data are presented as mean ± standard deviation (SD). Each experiment included three technical replicates per condition, which were averaged, and data from independent experiments were combined for analysis (*n* = 6 per group, from two independent experiments). Statistical comparisons were performed between titanium (Ti) and polymethyl methacrylate (PMMA) conditions. * *p* < 0.05.

Quantitative PCR was performed to assess relative mRNA expression of α-SMA, collagen I, FSP-1, and focal adhesion kinase (FAK) in corneal fibroblasts cultured on titanium and PMMA substrates. These markers were selected to assess fibroblast activation and material-driven mechanotransduction: α-SMA and FSP-1 indicate myofibroblast differentiation and fibroblast activation, respectively; collagen I reflects extracellular matrix deposition; and FAK mediates integrin-dependent adhesion signaling that links substrate properties to profibrotic responses. Expression levels of α-SMA were comparable between hCFs cultured on titanium and those cultured on PMMA (2.98 × 10^6^ ± 2.56 × 10^6^ for titanium; 4.4 × 10^6^ ± 2.62 × 10^6^ for PMMA; *p* = 0.3), with no statistically significant differences detected. Collagen I expression levels were also similar between the two materials (1.11 × 10^9^ ± 4.90 × 10^8^ for titanium; 1.24 × 10^9^ ± 6.61 × 10^8^ for PMMA: *p* = 0.7). In contrast, FSP-1 trended towards a higher mRNA expression in hCFs cultured on PMMA compared to titanium (5.90 × 10^7^ ± 1.39 × 10^7^ vs. 3.81 × 10^7^ ± 1.98 × 10^7^; *p* = 0.06). Similarly, FAK mRNA levels were significantly higher in the PMMA group than in the titanium group (1.50 × 10^7^ ± 5.21 × 10^6^ vs. 2.47 × 10^7^ ± 6.03 × 10^6^; *p* < 0.05) (Figure 4).

## 4. Discussion

The Boston Keratoprosthesis (BKPro) is the most commonly implanted artificial cornea and is indicated for use in cases of severe corneal pathology where a standard corneal transplant is unlikely to be successful. As an artificial cornea, the BKPro must be biocompatible with corneal tissue, integrating with the surrounding stroma with sufficient adherence at the device–tissue interface but with an appropriate level of inflammation and fibroblast activation so as to preserve corneal integrity, maintain stable epithelial coverage, and minimize the risk of complications such as corneal melt and retroprosthetic membrane formation that can compromise device retention and visual outcomes [1,2,6,7,8]. In the context of the BKPro, this definition of biocompatibility extends beyond cytocompatibility and encompasses how the backplate material (i) interacts with and promotes corneal stromal cell survival and growth, (ii) influences cell activation and wound healing responses, and (iii) contributes to long-term device–tissue interface stability [1,12].

The BKPro is available with a PMMA or titanium backplate, and both materials are well established as biocompatible materials. Previously, human corneal epithelial cell viability on PMMA versus titanium backplates has been evaluated, showing that titanium demonstrated superior corneal epithelial cytocompatibility compared to PMMA, supporting greater cell proliferation and reduced contact-dependent cell death [5]. In addition, human corneal fibroblast responses to titanium backplates with varying topographies have been evaluated, showing that smoother surfaces promote adhesion and organized matrix deposition while rougher surfaces suppress fibroblast proliferation and myofibroblast transformation, potentially mitigating retroprosthetic membrane formation [4]. In the present study, we compared how titanium versus PMMA influences human corneal fibroblast viability, proliferative capacity, profibrotic gene expression, and adhesion-mediated signaling markers relevant to device–tissue integration. This is a question with direct clinical relevance, both broadly with respect to BKPro biointegration and specifically regarding retroprosthetic membrane (RPM) formation, which is the most common complication following BKPro implantation [10,11].

We found that both titanium and PMMA supported human corneal fibroblast viability with comparable proliferation over time, consistent with prior reports demonstrating the cytocompatibility of these materials in corneal epithelial and stromal systems [3,4,5]. SEM analysis showed substrate-dependent differences at the cellular level, with our results indicating broader, more continuous surface coverage by human corneal fibroblasts on titanium compared to PMMA. Titanium surface characteristics, including microtopography and surface energy, have previously been shown to influence adhesion, proliferation, transformation, and matrix deposition of corneal cells [4,5]. These properties have been suggested to contribute to increased spreading and, therefore, to the higher spatial distribution observed on titanium in prior studies. In the present study, these observations likely reflect material-dependent differences in cell–substrate interactions, although specific physicochemical properties were not directly measured. In contrast, fibroblasts cultured on PMMA were sparsely distributed, occupying a smaller percentage of the available surface area. This distinctive cellular behavior may reflect not only surface topography but also material-specific biological effects, as prior in vitro studies have demonstrated that PMMA-based resins can reduce fibroblast viability and upregulate NFκB/NLRP3/IL-1β inflammatory signaling compared to alternative resin formulations, underscoring how biomaterial composition can directly modulate fibroblast activation and tissue–material interactions [18]. In parallel, BKPro-specific work emphasizes that native PMMA is relatively hydrophobic and inert, with relatively less intrinsic cell/tissue biointegration and weak collagen adhesion at the optic-graft interface [19]. These limitations can be partially mitigated through PMMA surface modification (i.e., plasma/chemical functionalization or hydroxyapatite nanoparticle immobilization) to increase hydrophilicity, improve collagen bonding strength, and support corneal stromal fibroblast adhesion and proliferation.

Immunohistochemical analysis showed that fibroblasts cultured on titanium expressed higher FSP-1 protein levels, whereas α-SMA expression was higher in cells cultured on PMMA.

While FSP-1 is commonly associated with fibroblast activation and mesenchymal responsiveness, it does not specifically denote myofibroblast differentiation, which is more accurately reflected by α-SMA expression [20,21]. Given that RPM tissue is characterized by activated fibroblasts and α-SMA-positive myofibroblasts [12,22,23], these substrate-dependent differences are mechanistically relevant. Titanium appears to favor a mesenchymal-associated phenotype, whereas PMMA may promote a more contractile, myofibroblast-like profile. Importantly, these phenotypic shifts occurred despite comparable viability, suggesting that material-driven modulation has an effect at the level of cell activation rather than cell survival.

Gene expression analysis provided further information on substrate-induced differences in hCFs. α-SMA and collagen I mRNA levels were comparable across substrates. FSP-1 showed a trend toward higher expression on PMMA, while focal adhesion kinase (FAK) transcripts were significantly upregulated in fibroblasts cultured on PMMA compared to titanium. FSP-1 is a calcium-binding protein expressed by activated fibroblasts that regulates cell motility and extracellular matrix remodeling, making it a marker of fibroblast activation [20]. FAK is a central mediator of adhesion-dependent and mechanosensitive signaling, linking integrin engagement to cytoskeletal tension and downstream profibrotic pathways [4,15]. In in vitro models, experimental evidence shows that FAK inhibition attenuates TGF-β1-mediated myofibroblast differentiation and reduces α-SMA expression [15]. Thus, elevated FAK transcription on PMMA suggests enhanced activation of adhesion-related signaling pathways at the material interface. Collectively, the differences between protein and transcript patterns suggest that substrate-dependent regulation occurs at multiple stages, involving post-transcriptional control and cytoskeletal protein stability. These phenomena have been recognized as contributing to discordance between mRNA and protein expression across biological systems [24]. While titanium seems to maintain a more widespread, FSP-1-leaning phenotype, PMMA appears to enhance adhesion-mediated signaling upstream of contractile activation, as evidenced by elevated FAK transcription and increased α-SMA protein expression. However, these interpretations are based on marker expression and transcriptional patterns and do not constitute direct evidence of functional pathway activation in the absence of targeted mechanistic or stimulation-based assays.

Accordingly, these findings should be interpreted as indicative of material-dependent modulation of fibroblast phenotype at multiple regulatory levels, rather than as evidence of a direct or linear relationship between gene and protein expression. Taken together, material composition influences not only fibroblast survival but also the migratory cytoskeletal organization and contractile, myofibroblast-like activation at the device-tissue interface, which are relevant to downstream tissue remodeling.

From a clinical perspective, RPM formation has been shown to represent a localized fibroproliferative response at the BKPro interface [1,12,22,23]. Although inflammatory mediators and cytokine gradients are key contributors [2,13,22,23], the mechanical microenvironment created by the backplate may influence how stromal fibroblasts interpret these signals [4,5]. Subtle differences in adhesion dynamics and mechanosensitive signaling could, over time, contribute to cellular responses relevant to membrane formation. In this context, the higher α-SMA level observed with PMMA in vitro suggests a shift toward a more contractile, myofibroblast-associated phenotype under these experimental conditions. Although a prior clinical study has reported higher rates of RPM formation with PMMA backplates than with titanium in certain cohorts, these observations vary across studies and cannot be directly inferred to the present findings [25]. Accordingly, the in vitro differences observed here should be interpreted as indicative of substrate-dependent modulation of fibroblast phenotype, rather than as direct evidence of differential RPM risk in vivo. It is also important to consider that, in vivo, corneal fibroblasts are not always in direct contact with the BKPro; rather, extracellular matrix and stroma are predominantly in contact with the device. It is also possible that the formation of myofibroblasts with their extracellular matrix-producing properties could potentially be beneficial for device-tissue integration and wound healing. Thus, the present findings should not be interpreted as evidence of the superiority of one material over another, nor as establishing a causal relationship between backplate material and clinical RPM incidence, but rather as demonstrating that, under these controlled in vitro settings, backplate composition can modulate stromal biology at the cellular level in ways that may be relevant to tissue remodeling processes.

Consideration should also be given to the fact that this study was conducted in a simplified in vitro monolayer culture system that may not fully recapitulate the three-dimensional architecture, extracellular matrix composition, mechanical forces, and inflammatory milieu present at the BKPro–tissue interface in vivo. The selected marker panel (FSP-1, α-SMA, COL1A1, and FAK) was chosen to capture complementary aspects of fibroblast activation, myofibroblast differentiation, extracellular matrix production, and mechanosensitive cell-material interactions. However, this panel does not fully encompass inflammatory signaling pathways, which warrant further investigation. Also, a discrepancy between FSP-1 protein and transcript expression was observed across substrates. While differences between mRNA and protein levels are recognized in biological systems, the present study does not allow the determination of the underlying mechanisms. This finding should therefore be interpreted as an observation and represents an area for future investigation. Corneal fibroblasts were evaluated under baseline conditions without profibrotic or inflammatory stimulation, and substrate-dependent differences may differ in a cytokine-rich postoperative environment. Additionally, while immortalized human corneal fibroblasts enable experimental consistency and reproducibility, they may not fully recapitulate the phenotypic heterogeneity, differentiation capacity, and stimulus responsiveness of primary stromal cells, thereby limiting direct extrapolation of these findings to in vivo conditions. Also, this study focused only on stromal fibroblasts, whereas RPM formation is multifactorial and involves additional cellular contributors [12]. Accordingly, these findings provide mechanistic insight into material-cell interactions but should not be interpreted as predictive of clinical outcomes, including RPM incidence or severity.

## 5. Conclusions

Titanium and PMMA both support and exhibit cytocompatibility with human corneal fibroblasts. However, these substrates differentially influence corneal fibroblast organization, activation marker expression, and adhesion-related gene regulation. By linking biomaterial properties to clinically relevant stromal activation markers in the BKPro microenvironment, this study provides a mechanistic framework connecting material composition to the host response at the BKPro–tissue interface. Further studies in more physiologically relevant models will be necessary to determine how these substrate-dependent cellular responses translate into long-term clinical outcomes. As such, the present findings reflect modulation of fibroblast behavior under baseline conditions and should be interpreted within this context, as responses may differ in the presence of pro-fibrotic or inflammatory stimuli that shape cellular behavior in vivo.

## Data Availability

The data presented in this study are available on request from the corresponding author due to privacy.
